# Head and Neck 3D Bioprinting—A Review on Recent Advancements in Soft Tissue 3D Bioprinting and Medical Applications

**DOI:** 10.3390/jfb16070240

**Published:** 2025-06-30

**Authors:** Iosif-Aliodor Timofticiuc, Ana Caruntu, Christiana Diana Maria Dragosloveanu, Andreea-Elena Scheau, Ioana Anca Badarau, Argyrios Periferakis, Serban Dragosloveanu, Andreea Cristiana Didilescu, Constantin Caruntu, Cristian Scheau

**Affiliations:** 1 Department of Physiology, The “Carol Davila” University of Medicine and Pharmacy, 8 Eroii Sanitari Boulevard, 050474 Bucharest, Romania; 2 Department of Oral and Maxillofacial Surgery, “Carol Davila” Central Military Emergency Hospital, 010825 Bucharest, Romania; 3 Department of Oral and Maxillofacial Surgery, Faculty of Dental Medicine, Titu Maiorescu University, 031593 Bucharest, Romania; 4 Department of Ophthalmology, Faculty of Dentistry, The “Carol Davila” University of Medicine and Pharmacy, 020021 Bucharest, Romania; 5 Department of Ophthalmology, Clinical Hospital for Ophthalmological Emergencies, 010464 Bucharest, Romania; 6 Department of Radiology and Medical Imaging, “Foisor” Clinical Hospital of Orthopaedics, Traumatology and Osteoarticular TB, 021382 Bucharest, Romania; 7 Akadimia of Ancient Greek and Traditional Chinese Medicine, 16675 Athens, Greece; 8 Elkyda, Research & Education Centre of Charismatheia, 17675 Athens, Greece; 9 Department of Orthopaedics and Traumatology, The “Carol Davila” University of Medicine and Pharmacy, 050474 Bucharest, Romania; 10 Department of Orthopaedics, “Foisor” Clinical Hospital of Orthopaedics, Traumatology and Osteoarticular TB, 021382 Bucharest, Romania; 11 Department of Embryology and Microbiology, Faculty of Dentistry, The “Carol Davila” University of Medicine and Pharmacy, 050474 Bucharest, Romania; 12 Department of Dermatology, “Prof. N.C. Paulescu” National Institute of Diabetes, Nutrition and Metabolic Diseases, 011233 Bucharest, Romania

**Keywords:** 3D bioprinting, head and neck, soft tissue, bionics, tissue engineering, physiology, vascularized scaffolds

## Abstract

Recent developments in 3D bioprinting offer innovative alternative solutions to classical treatments for head and neck defects. Soft tissues in an anatomical area as diverse in composition as the head and neck are complex in terms of structure and function. Understanding how cellular interaction underlies functionality has led to the development of bioinks capable of mimicking the natural morphology and roles of different human parts. Moreover, from the multitude of recently developed materials, there are now many options for building scaffolds that potentiate the activity of these cells. The fidelity and accuracy of the utilized techniques ensure maximum precision in terms of model construction. Emerging technologies will allow for improved control of the scaffold, facilitating optimal results in the treatment of various pathologies, without concerns about the availability of donors, immunological response, or any other side effects that traditional treatments withhold. This paper explores the current landscape of bioprinted scaffolds and their applications in the head and neck region, with a focus on the properties and use of natural and synthetic bioinks in the attempt to replicate the biomechanical features of native tissues. Customization capabilities that support anatomical precision and biofunctionality are also addressed. Moreover, regulatory requirements, as well as current challenges related to biocompatibility, immune response, and vascularization, are critically discussed in order to provide a comprehensive overview of the pathway to clinical application.

## 1. Introduction

The head and neck region presents high complexity, mainly due to the vast vascular and neural networks integrated into a biomechanical system that ensures a multitude of functions, including sensorial and motor control [1,2]. The soft tissue components of this region play an important role in the integrative sensorial system, which highly contributes to the adaptation of humans in their natural environment [3]. The system consists of complex organs and supporting elements [4,5].

Defects in this anatomical region, with its numerous structures with complex functionality, can greatly impact patients’ lives, either psychologically or physically. Causes of these defects can include oncologic resections, other surgical interventions, congenital pathologies, genetic disorders, or post-traumatic tissue loss [6,7,8]. Most times, the complexity of treatments requires a multidisciplinary approach to restore the functionality and aesthetics of the affected elements. Increasingly complicated transplantations are attempted, but functional results can still be further improved, and supplementary rehabilitation methods are required [9,10,11]. Recent research focuses on the development of new technologies and treatments aimed at individualized solutions with optimized outcomes for patients.

3D bioprinting is an interdisciplinary branch of the 3D printing industry developed specifically for medical applications [12]. Numerous applications already exist, and the potential of the field is ever-increasing [13,14,15,16,17]. The materials used in this field are carefully designed in terms of mechanical and bioactive properties and include mixes with added cultured cells, also known as bioinks [18,19]. Therefore, while maintaining cell viability and compatibility in a specific material, 3D models or scaffolds are built to serve precise functions mimicking physiological functions [18,19]. Numerous types of technologies have been developed to serve the purposes of 3D bioprinting, such as micrometer precision, controlled deposition of cells, high functionality, increased viability, and bioactive properties [20,21].

The most relevant methods developed in 3D bioprinting are (1) droplet-based bioprinting, composed of inkjet-bioprinting and laser-induced forward transfer, which ensures high resolution and fast printing due to the controlled cell deposition using droplets of cell-laden biomaterial [22]; (2) extrusion-based bioprinting, highly used in current studies due to a large range of available biomaterials with layer-by-layer printed bioinks [23]; (3) laser-assisted bioprinting, which emerged from droplet-based bioprinting [24]; and (4) stereolithographic bioprinting, which uses photopolymerization, similar to digital light processing (DLP) or stereolithography (SLA) 3D printing techniques [25].

**Figure 1:**
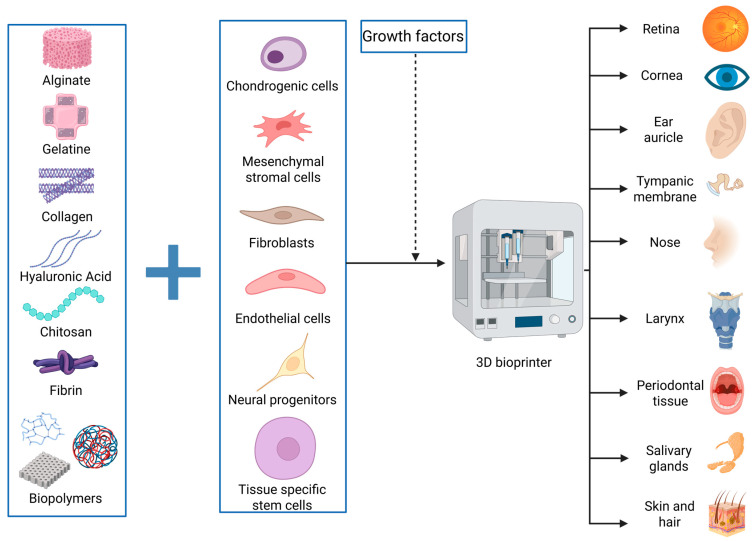
Summary of the results obtained by bioprinting soft tissues of the head and neck. Relevant materials or cells used in this field are presented. Created in BioRender. Timofticiuc, I. (2025) https://BioRender.com/e6j0vbk. Date of last access—3 May 2025.

One of the advancements desired by the medical and engineering industries is limiting the use of human donors for patient treatments and creating parts that can mimic the functions of various human elements with high specificity. The simpler term for this is creating bionic parts. Bionics is a field based on building elements that can mimic the form or function of something that already exists in nature to serve human evolution and development [26]. Adapting this definition in our paper, we highlight the recent therapeutic advancements made possible by 3D bioprinting, with inspiration from human parts and their functionality (Figure 1). Alongside presenting and integrating the achievements of 3D bioprinting in head and neck soft tissues, we focus on the main challenges and drawbacks of this method, while exploring potential workarounds and future perspectives.

## 2. Head and Neck Soft Tissue 3D (Bio)Printing

The main defects affecting different parts of the head and neck region are reviewed below, highlighting the limitations of traditional treatments. 3D bioprinting represents an opportunity for the development of specific treatments, as well as the supply of, theoretically, an unlimited amount of biocompatible tissue. In current practice, when designing and building soft tissue scaffolds, the mechanical characteristics are extremely important. Presently available biomaterials, as developed and described in recent studies, may be incorporated into bioprinted elements meant to replace soft tissues of the head and neck. The similitude in mechanical properties to the anatomical structures targeted for restoration is one of the strong arguments in this regard (Table 1 and Table 2).

The head consists of numerous complex components, with structural, sensitive, motor, or sensorial roles. Bioprinting is a great option due to the possibility of creating high-precision models and multilayered constructs, where various types of cells can be introduced differently in each layer of the models, thus creating more precise builds that can mimic the natural tissues or organs more closely [130,131,132]. In the following sections, we highlight the main advancements in the 3D bioprinting of eyes, ear components, nose, salivary glands, oral parts, skin, and hair, while addressing the main limitations and potential solutions to current problems. Vascularization and neural control are critical aspects for fully functional models and will be discussed in the final section of this paper.

### 2.1. Corneal 3D (Bio)Printing

Considering all the possible causes of blindness worldwide, corneal opacity stands out as a distinct subgroup. Even though such causes represent less than 5% of the defects that lead to blindness, many of them, such as a number of diabetes-induced corneal changes [133], are treatable, and corneal transplantation is the most used method to achieve full vision recovery [134,135]. The transplantation techniques have evolved, from large and multilayered grafts in 1956 to single-layered grafts or partial grafts in the 21st century, offering safer and faster recovery. However, the number of patients in need far exceeds the number of donors, with a current ratio of approximately 70 to 1 [134,136].

Despite donor shortage, there exist alternatives, and the most promising for the future of corneal repair is the FRESH method, a 3D bioprinting protocol used by Isaacson et al. in 2018 to build the first fully 3D bioprinted corneal stroma [137]. The challenge in 3D printing a corneal stroma is obtaining good transparency and optimal architecture of the build, thus ensuring all the physical phenomena of image formation [138].

A common mixture used in corneal 3D bioprinting is based on three materials: collagen, alginate, and gelatin [139,140]. Collagen is used as a material in 3D printing and does not present ideal properties; collagen-based builds perform weakly in terms of mechanical behavior; however, in combination with other transparent materials such as alginates and gelatin, the performance can be improved, especially in cornea bioprinted constructs, where transparency is essential [139,141,142,143].

Unexpectedly, utilizing three transparent materials does not necessarily result in a transparent final 3D-printed product. Several attempts in the last 10 years showed that transparency is perhaps the hardest characteristic to achieve. The reasons for this significant drawback appear during the printing process. Due to the complicated dome shape of the cornea, the 3D printed layers collapse and create micro gaps filled with air or with insufficiently processed biomaterial, thus affecting light transmittance due to opaque spots or gaps with differing refractive indices [140].

A team of researchers addressed the main challenges of 3D printing corneal parts, i.e., the high costs, the difficulty in printing with viable cells, and the low transparency. A partial thickness corneal construct using a low-cost modified 3D printer was built. Optimal transparency was achieved using a concave mold as support to prevent layers from collapsing, therefore moving away from the FRESH technique, which created porous builds often filled with air bubbles [140].

From our perspective, this method, which was only used for partial grafts, can be scaled up to full corneal constructs. However, scaling up could bring other technical issues due to the printing process, increasing costs. Furthermore, the team did not use viable cells in this method, relying on the post-operative cellularization of the construct—an aspect that might not be as feasible in full corneal stroma 3D-printed builds [140].

An example that highlights the need for viable cells and very detailed techniques in 3D bioprinting large models can be seen in a study where a cell-laden corneal stroma was printed. Using a combination of technologies and Human Corneal Keratocytes (HCKs), full corneal stroma equivalents were able to ensure optimal transparency (75–90%) while maintaining their mechanical properties and integrity [139].

Notably, the FRESH method was used with the same three transparent materials mentioned before, but the performance was improved by combining 3D bioprinting, micro-transfer molding, and optimized bioinks [139]. In our opinion, this study highlights the importance of sophisticated techniques and the use of viable cells, ensuring a physiologically similar microenvironment that could maintain the viability of the construct over time.

However, issues still remain regarding the in vivo interaction between biomaterials and viable cells, which might have uncertain consequences [139]. Under that consideration, it was observed that using different materials, such as a decellularized extracellular matrix/gelatin methacryloyl (CECM-GelMA) bioinks, a corneal matrix with no viable cells and with no support structure can be built without affecting the transparency, nor the structural integrity of the construct [144]. It was demonstrated that the mix of substances in this bioink provides attachment sites for patient cells while also reducing inflammation [144]. However, it is uncertain how the fourth dimension affects these types of builds, and a major challenge is determining how long the 3D-printed corneal stroma equivalents can perform optimally. Furthermore, it is essential to know whether replacement is possible once defects appear, and more importantly, how many times these revisions can be performed.

### 2.2. Retina 3D (Bio)Printing

The retina presents a complex architecture [145]. To understand the possibility of 3D bioprinting a retinal equivalent, we must first address the main challenges from the perspective of printing technology and the availability of printable materials. It is known that the retina has a multilayered structure, with various types of cells [146]. From this observation, we can see two conditions that need to be fulfilled in the final 3D construct: (1) bioprinting the cells in an extracellular matrix (ECM) equivalent similar to the retinal microenvironment in terms of chemical, physical, and physiological properties; and the necessity of (2) designing a heterogeneously multilayered build.

#### 2.2.1. Bioprinting Retinal Cells and ECM-Equivalent Conditions

The cellular composition of the retina is mainly made up of photoreceptors (PRs), horizontal cells, bipolar cells, amacrine cells, and ganglion cells [147]. The first step for an optimal bioprinting process is ensuring the cell’s availability and ability to survive, both in vitro and during the mechanical stress of the printing phase. PRs can be obtained by differentiation from retinal progenitor cells, but the cells cannot survive isolated in vitro [148]. Therefore, PRs require the assistance of the retinal ECM composition to develop and survive in vitro [149].

It was demonstrated that maintaining the viability of PRs is difficult in vitro with ECM equivalents that do not mimic the physical properties of the retinal ECM [148]. The retinal microenvironment contains mainly hyaluronic acid [150]. A team of researchers tried to achieve an environment suitable for photoreceptor development using methacrylate hyaluronic acid [148]. They managed to achieve the necessary stiffness for PR development (10–20 kPa) and enhanced cell viability and development by adding retinal pigment epithelium to complement the HA-based gel [148].

In our view, bioprinting should be developed towards the possibility of building structures that require as few viable components as possible, to provide real and sustainable advantages in clinical applications. An interesting avenue is the possibility of bioprinting the retinal pigment epithelium (RPE), a component known to offer optimal circumstances for apoptosis, and for physiological activities of retinal cells [151]. Due to a paucity of studies where other components are used for PR bioprinting, RPE remains, at least for now, the only solution that transforms the artificial ECM equivalent suitable for retinal bioprinting.

RPE consists mainly of lipids (>50%) [152]; thus, bioprinting these substances represents the next main step to be taken for the future of bioprinting RPE. Lipids, mainly because of their thermoplastic behavior, are not yet suitable for the most commonly used bioprinting methods, especially those that use layer-by-layer technology [153]. However, there is a possibility to adapt lipids to 3D bioprinting by using droplet-based deposition as the main form of “layering” [153]. That being said, from our perspective, the faster way to achieve optimal PR bioprinting is by advancing the bioprinting of RPE, rather than isolating or developing other biomaterials that could allow the survival and development of PRs.

Regarding the other cellular components of the retina, data is scarce regarding the 3D bioprinting of functional parts of the eye. The steps needed to achieve 3D printability in these types of cells, thus completing the structure of a retina in regard to cellular composition, are, in our view: (1) obtaining and developing amacrine cells, horizontal cells, and ganglion cells in vitro; (2) testing the mechanical stress they can sustain; (3) identifying or developing biomaterials that support their viability; (4) isolating a 3D bioprinter compatible with the obtained mixture; (5) developing scaffolds viable for an undetermined period; (6) creating solutions to anatomically and functionally interconnect these cells; and (7) connecting the cellular network with the blood supply and ensuring neovascularization.

#### 2.2.2. Designing a Heterogeneously Multilayered Build

Building 3D scaffolds using bioinks for drug testing was a major achievement, which ensured a better understanding of how different substances interact with the retinal microenvironment [154,155]. In an apparently unconnected line of research using other cellular lines (Y79, ARPE 19), for a scaffold used for drug testing, researchers also revealed the possibility of building multi-layered constructs with different cell types in the composition of each layer—one basal layer of ARPE 19 cells topped up with two distinct layers of Y79 cells [154]. This breakthrough opened numerous possibilities for developing multilayered builds in other applications.

However, the ultimate goal is to build a structure able to mimic its living counterpart. One of the options is to design superimposed cell-laden hydrogel layers. However, this comes with many challenges. To start with, due to the high density of cells in a viscous surrounding, the shear stress increases dramatically, which affects cell viability [156]. In addition, the layers can often lack uniformity, hence mimicking tissue behavior becomes extremely difficult [156]. The other solution in constructing multilayered scaffolds is by printing the cells without the surrounding hydrogels, thus allowing the cells to create their ECM [156]. Nevertheless, drawbacks occur when the layers with different types of cells need to be stabilized to form a mechanically resistant structure [156].

It is reasonable to consider that a solution could reside in building a structure with layers consisting of highly porous degradable biomaterials, thus ensuring the stability of the structure and the position of the cells in the scaffold, with the ECM produced by the cells replacing the degradable artificial surroundings over time. Adopting this mixed solution could support the mechanical behavior in the initial phase of the print. A porous construct could help preserve the viability of the cells by reducing the mechanical stress, while also offering them space to produce their own ECM. By degrading over time, the “natural” ECM could ultimately fully replace all artificial environments in a controlled manner.

### 2.3. Exploring the Feasibility of Developing a Bionic Eye

**Figure 2:**
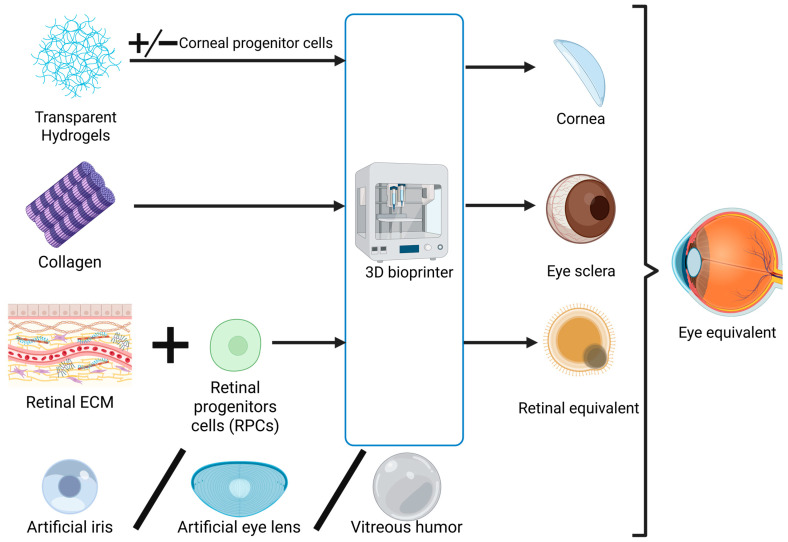
Schematic representation of the process of 3D bioprinting eye components and their integration in an eye equivalent (bionic eye). Created in BioRender. Timofticiuc, I. (2025) https://BioRender.com/d6q5va5. Date of last access—21 June 2025.

In essence, the components of the eyeball have the highest complexity in terms of bioprinting. In order to predict the possibility of producing a bionic eye in the future (Figure 2), we must analyze the possibility of printing the other components indispensable for ocular functions and consider their integration into a functional and interdependent system. The structures requiring replication in the outer layer of the eye are the sclera, a fibrous tissue, and the cornea, which is covered by the conjunctiva as a transparent membrane [157]. Moving to the middle layer, the iris, ciliary body, and choroid are structures that are in continuity and functionally connected with each other [157], which may be affected together in some pathologies, like cancer [158]. The retina, the innermost layer, has been discussed in extenso above [157]. Additionally, solutions equivalent to the vitreous humor, aqueous humor, and crystalline lens need to be addressed [157].

Regarding the iris and scleral constructs, data is very scarce on bioprinting or 3D printing studies in which models are built that result in a final product with at least minimal functionality. However, there are a few reports of ocular prostheses created to fulfill an aesthetic role, where realistic sclera and iris were produced [159,160].

The sclera is essential for ensuring the biomechanical properties of the eyeball, such as maintaining intraocular pressure for optimal image formation and light transmission [161]. However, from a metabolic standpoint, the sclera does not exhibit activities essential to the main function of the eye. Therefore, in 3D printing a sclera, only mechanical performance is essential [161]. The main component of the sclera is collagen (28% of wet weight, with water comprising 68%), with collagen type I representing around 95% of all types of collagens found in the sclera [161,162]. A 3D-printed sclera needs to be able to withstand pressures of around 12.8 ± 2.7 mm Hg, the mean value for intraocular pressure, requiring an optimal modulus of elasticity around 750 kPa [163]. Collagen type I bioprinting is already possible, but the achieved elasticity is around 200–250 kPa [164]. A collagen ink was also used to create a high-resolution print containing type III collagen, with a resulting elasticity modulus reaching only 679 kPa [165].

These findings are good explanations for why limited advancements have been made in 3D printing a mechanically functional sclera. However, newly developed materials and mixtures could balance the stiffness and the elasticity needed for a potential sclera construct. In our view, a solution for reaching high elasticity while also preserving structural strength could involve perfecting a printing method for collagen type I with improved mechanical features (which will be closer to mimicking the real structure of the sclera) or printing collagen type III in mixtures with an elastic biomaterial. One example in this direction could be MeTro, a recombinant human tropoelastin modified by adding methacryloyl groups, thus resulting in a substance that enhances elasticity but also provides mechanical stability [166].

With regard to the iris or the crystalline lens, there are already available methods that do not necessarily involve 3D bioprinting but require a process of manufacturing that has already proved highly efficient for patients. Some examples in this direction include the silicone iris prosthesis ArtificialIris (HumanOptics, Erlangen, Germany), used as an innovative option for the treatment of various types of iris defects [167], and artificial lenses used in cataract surgeries [168,169]. Given that these are already highly functional and cost-efficient approaches for replacing the iris and the lens, bioprinting them would not bring additional value and could be considered of lesser importance.

The vitreous humor plays a crucial role, in complementarity with the sclera, in the mechanical strength of the eye. It also plays an important role in light transmittance, having a refractive index of 1336, similar to that of the cornea [170]. The vitreous humor consists mainly of water (approximately 90%), but also contains important biomechanical components such as collagen type II and hyaluronic acid [171,172,173]. Both hyaluronic acid and collagen type II are printable [164,174], so a printed vitreous humor could be developed if the kinematic viscosity of 300–2000 cSt could be reached [175]. The challenge is ensuring a higher viscosity in the central part of the humor [170].

In our view, the flow of aqueous humor in the eye is most probably not achievable, due to the challenges of ensuring drainage and exchange of nutritional substances [176]. Using a biomaterial rich in water and glucose that would also maintain transparency and optimal transmittance of light could be the solution, but, to our knowledge, no reported materials fit this description [177].

Finally, after addressing the main components of the eye and the possibility of building them using 3D printing, we still have to face the main challenge, which is integrating all the structures described above in an interdependent system and ensuring their supply of nutrients and oxygen via vascularization. These represent drawbacks not only for the bionic eye but for any artificially-made functional organ. Further research into understanding and controlling neovascularization, as well as developing bioinks with VEGF-stimulating materials, may offer solutions for the future of bionic organs. As such, the possibility of printing choroid or conjunctiva remains more distant, due to their heavy reliance on vascularization [178,179].

### 2.4. Ear Auricle 3D Bioprinting

In order to print an auricle or pinna that mechanically and metabolically mimics a human ear, a stable and bioactive cartilaginous structure first needs to be obtained [180]. The outer ear plays an important role in the formation of sounds due to its complex shape and consistency [181]. Therefore, besides the need to create an optimal turnover of the cells contained in the final print, the shape and the mechanical properties are also very important. There are numerous approaches for creating a stable bioink that serves the purpose of creating a chondrogenic scaffold. A group of researchers developed a new bioink by generating ECM derived from microtia tissue [182]. The affected human ear was implanted subcutaneously in mice, and from that, a microtissue was developed, which further became the source for the cells contained in a bioink used in the 3D bioprinting of a normal ear [182]. Hence, the team managed to develop a bioink capable of creating a model that performed well in vivo while also presenting great mechanical properties, such as optimal stiffness and elasticity [182]. However, due to its invasive character, other methods need to be identified. An innovative technique was developed in this regard, called Digital Near-Infrared Photopolymerization (DNP)-based 3D printing. Relying on the properties used in DLP or SLA, such as photopolymerization under UV light [174], but using Near-Infrared Red light, which is more deeply penetrating, it became possible to induce polymerization processes in the subcutaneous layer of a mouse, thus making it a non-invasive method [183]. With this approach, the research team created a human ear model under the skin of a mouse by injecting the animal with hydrogel mixed with chondrocytes. The light was directed using a digital micromirror device to activate the polymerization process. The next step would be to extract the metabolically active ear from the animal model and surgically attach it to the patient. At this stage, however, a drawback appears: the cells added to the build require resources to ensure proper turnover, and methods for ensuring cell viability need to be developed before transitioning to patient trials.

Considering that the cartilaginous tissues are avascular [184], a good approach to ensuring nutrients and oxygen supply might be creating a highly porous structure, which can then be connected to the patient’s vascularization. Furthermore, to ensure good aesthetic final results, the final build should be covered with bioprinted skin.

Another important aspect of the possibility of creating accessible bioprinted prostheses is the cost, and creating more accessible printers could be the solution to this issue. For example, by only making slight modifications to a classical thermoprinter, an outstanding bioprinter was obtained and used to print an equivalent of a human bioactive and cell-laden ear [185].

One of the areas where further optimization would be beneficial is the materials used for creating the cell-laden scaffold with chondrogenic properties. It was shown that stem cells and chondrocytes require soft materials for optimal development, a feature which appears to contradict the implicit rigidity and hardness of the final product [186]. Good results can be obtained by mixing hydrogels with other bioactive materials such as hydroxyapatite, but further research in the area of developing other materials is needed [186].

Another challenge in the generation of cartilaginous structures is the limited time during which chondrogenic-promoting cells maintain their activity and properties [186]. Human mesenchymal stem cells or human chondrocytes are commonly utilized in creating cartilage, but they mostly lose their chondrogenic properties after the fifth passage [187,188,189]. A solution that could be attempted is the usage of human amniotic fluid-derived stem cells, which can maintain their chondrogenic properties for a longer time and are also bioprintable [190,191].

To summarize, while great results have been reported in bioprinting human-like outer ears, the possibility of applying these constructs in humans remains minimal and costly, even if achievable. Furthermore, the currently existing biomaterials and utilized cells do not yield consistently effective results, indicating that additional research is required.

### 2.5. Tympanic Membrane 3D (Bio)Printing

The most common tympanic membrane defects are perforations [192]. Around 55,000 surgeries are performed annually and are mostly represented by tympanoplasty [193]. The success rate reported in tympanoplasty surgery varies from under 50% to up to 100% in some cases [194]. These particular statistics suggest that this kind of surgery is strongly dependent on the dimension and shape of the defect, but also on the practical abilities of the surgeon. The main challenge for surgical teams is related to the hand-crafting of grafts, and in cases of unusual morphologies of tympanic membrane defects, the results can be less predictable [195].

Bioprinting patient-specific grafts for defects of the tympanic membrane is now a suitable option for modern tympanoplasty, with a higher success rate. A team of researchers explored this direction by printing GelMA implants, which were later tested in vivo on animal models. An accurate model was built after scanning the tympanic lesion, with precise dimensions and shape. Supportive “butterfly” structures for anchoring the grafts without sutures were also designed [194]. The team reported the achievement of a construct with good mechanical properties, and the supportive structures were able to anchor the grafts even at pressures above 5 kPa [194].

However, it is known that the human tympanic membrane, similar to those of other mammals, can withstand an ultimate stress of over 1MPa [196,197]. Although the possibility of applying patient-specific grafts with no sutures is innovative and opens new possibilities, the mechanical properties could be further improved. One solution to increase the tolerated stress of materials compatible with 3D printing involves decreasing the spacing between filament lines contained in the final product [198]. Therefore, by adding different biomaterials to the final mixture or adapting the temperature or light exposure time, reinforced hydrogels can be obtained to withstand mechanical stress as high as 0.4 MPa [198].

There are a few reported studies where reinforced hydrogels were obtained, but their builds exhibit characteristics that deviate from the optimal mechanical and vibratory properties of the eardrum. For example, a tympanic membrane made of PCL, a material with great elastic and mechanical properties, managed to withstand forces of around 142 kPa [199]. Although this value is much more suitable for behaving like a real eardrum, a graft three times thicker (300 µm) than the physiological thickness of the tympanic membrane was required, which can seriously affect the elastic properties of the eardrum [199].

To address the challenge of implementing elasticity and optimal vibrational properties in the graft, a different approach was needed. As suggested above, reinforced hydrogels can be the perfect candidate to obtain desirable mechanical properties. Thus, a scaffold consisting of three polymers (PLA, PCL, and polydimethylsiloxane-PDMS) was built and filled post-printing with hydrogel. The construct exhibited superior features, both acoustically and mechanically [200]. The final thickness of the graft was around 700 µm—seven times thicker than the human eardrum; however, by designing a radial scaffold with large areas that were later filled with a thin layer of <50 µm of hydrogel, the authors managed to overcome the low elastic properties of other grafts [200]. Still, due to the high density of the graft, acceptable acoustic results were reported only between 1000 and 6000 Hz, while under 1000 Hz, sound transmission dropped significantly [200].

Another solution to reinforce hydrogels for better mechanics was the addition of KerMA (methacrylated keratine) to the printing inks [201]. Considering that keratine presents good mechanical properties, the designed grafts were able to withstand stress up to 0.37 MPa [201]. Furthermore, this is the only study, to our knowledge, in which the graft could be filled with human stem cells, making it a promising approach and the first with reported results regarding its bioprintability [201].

Printing an eardrum is a very demanding task due to its viscoelastic and mechanical properties. While numerous advancements have been made, no constructs performing as well as a human eardrum have been developed. Printing grafts specific to the dimensions of the defect could optimize the surgical time and complexity, but significant scientific improvements are still required in this regard. As previously discussed, further development of printing technologies may allow for more accurate and thinner structures while preserving functional mechanical strength.

Furthermore, none of the articles reviewed here report the elastic modulus of their constructs, an aspect that, in our view, is important for future research, given that the tympanic membrane has a Young’s modulus between 34 and 59 MPa [202]. Additionally, hybrid approaches could represent potential solutions, for instance, continuing the clinical use of cadaveric tympanic membranes while reducing the risk of infection by coating them with nanoparticles filled with antibiotics [201].

### 2.6. Nose 3D (Bio)Printing

This section addresses the possibility of manufacturing a bionic nose. There are three main components of interest, indispensable for a minimal function of a nose equivalent: the cartilage, the nasal epithelium, and the olfactory system sensitive to odors [203]. The nose cartilage plays a double role: cosmetic and functional, i.e., ensuring the optimal velocity and flow of air for good temperature regulation, allowing enough time for the interaction of receptors and air particles, as well as air filtration [204].

Several bioinks have been researched to mimic the nose cartilage. One of them stands out due to the approach used for crosslinking the hydrogels—horseradish peroxidase added to a mix of living cells, glucose, alginates, and cellulose nanofibers [205]. The innovation lies in the function of the peroxidase to catalyze conjugation reactions, thus ensuring optimal crosslinking of gels without altering cellular viability [205]. Cellulose nanofibers have the advantage of superior control of the bioink viscosity, a characteristic relevant both to ensuring an optimal printing process and to the development and survival of the cells [206,207].

Furthermore, this type of nanofiber can mimic the structure of cartilage collagen, enhancing its mechanical stability. However, when relying only on alginates and nanofibers, higher concentrations are required in order to obtain such a complex and robust structure as the nose [205]. These particular aspects directly affect cell viability, limiting survival to one week—drawbacks compounded by the already increased cytotoxicity induced by the H_2_O_2_ generated by the peroxidase [205].

Other ways to enhance the robustness of the final build are needed. An alternative could be the use of GelMA as the main scaffold material, a gel that ensures high mechanical stability without increasing the early death rate of the cells added to the bioinks [208]. PEGDMA (polyethylene glycol dimethacrylate) can also be added to the mix, with a two-fold advantage: increasing the softness of the 3D constructs and ensuring a suitable cell attachment with prolonged viability [209,210].

Other biocompatible materials can be used as equivalents to the nasal cartilage, and collagen type I is a great candidate [211]. However, when choosing optimal mixes for bioprinting cartilages, four main aspects must be considered: (1) using low temperatures that the cells can tolerate while ensuring good printability; (2) designing or creating pores with different biomaterial concentrations to support cellular viability; (3) obtaining a balance for the needed mechanical stability without affecting the cells during printing or afterward due to increased stiffness; and (4) decreasing viscosity as much as possible without affecting the deposition of the materials in the final constructs, thereby ensuring proper cell development and prolonged viability [212,213,214].

Another crucial contributor to the functionality of the nose is the nasal epithelium. Its roles include filtering harmful particles, creating a barrier against pathogens and other antigens or allergens, and secreting mucus to support the aforementioned properties. An artificial nasal epithelium was created to meet these characteristics [215]. Using human nasal progenitors, five major epithelial cell populations were obtained: basal, suprabasal, goblet, club, and ciliated cells [215]. This multilayered development of various cell types was obtained with the use of droplet-based bioprinting [216]. It was postulated that this technique minimizes the mechanical stress typically experienced by cells during layered printing, thus supporting proper epithelial architecture development [215].

A main focus of recent literature has been obtaining individual parts of different organs. However, the necessity of creating a scaffold for their attachment is often left out. This could represent a good direction for future research, specifically for attaching the nasal epithelium to the nasal cartilage. A viable solution could be a scaffold able to produce ECM, while also maintaining the viability of the cells on both sides, i.e., the chondrocytes in the nasal cartilage and the epithelial cells in the mucosa equivalent. Therefore, researching a good candidate for this type of ECM where the TGF-β1 concentration is controlled is needed [217].

Developing a system that can perceive the sense of smell through bioprinting can be a difficult task due to the complexity of the neuronal connections and the receptive system [218]. Digital sensors already exist, which are usually designed on top of biocompatible polymers that are used in experimental settings [208]. However, numerous challenges remain. Cellular viability in the aforementioned studies was not tested beyond 6 weeks, so it is unclear what the cellular behavior will be, especially when interacting with the biocompatible scaffold. Given the presence of metabolic activity, there is no alternative to “clean” the viable cells in situ without properly developing neovascularization in the scaffold. Additionally, it is uncertain whether designing bionic organs in a hybrid system with digital parts will create any immunological responses or increased cytotoxicity in the scaffold itself. These are a few aspects that need to be further researched to get closer to the goal of replacing human body parts.

### 2.7. Periodontal Tissue 3D Bioprinting

The periodontium protects and supports the teeth, and is formed of two soft tissues (the gingiva and the periodontal ligament) and two hard tissues (the alveolar bone and the cementum) [219]. Diseases affecting the periodontium are considered important oral pathologies; periodontitis in particular affects 1 in 5 adults [220,221]. Left untreated, periodontitis leads to tooth loss [222], and the chronic inflammation does not allow a proper integration or sustainability of dental implants, if used as a treatment for tooth loss [223]. In contrast with traditional, non-surgical treatments of periodontitis, recent studies have employed a treatment based on stem cell-based regeneration of the periodontal tissues [224,225,226,227,228].

Proper proliferation and bioactivity of stem cells can be achieved through incorporation in biomaterials-based scaffolds via 3D bioprinting. As discussed in earlier sections of this paper, GelMA and sodium alginate mixes are frequently used in bioprinting applications due to their great compatibility with printers. In a study on periodontal tissue regeneration, bioactive glass microspheres, growth factors, and stem cells were added to the biomaterials mix in an attempt to simulate the ECM bioactive properties of the periodontium [229].

The glass microspheres promote osteogenesis and angiogenesis, while the GelMA-sodium alginate hydrogel simulates the mechanical properties of the periodontium, allowing stem cells to reconstruct both soft and hard tissues [230,231,232,233,234]. Repairing both types of periodontal tissues at the same time has proven challenging via traditional methods and even through novel approaches using only stem cells and hydrogels. By adding growth factors such as BMP-2 and PDGF, adequate simultaneous healing was achieved [229]. Although the mix of substances was crucial for fast healing, optimal water retention, and metabolite flow, the researchers reported low mechanical strength of the scaffold when tested in vivo on beagle dogs. Thus, an alternative to the utilized hydrogels needs to be tested in order to improve the final mechanical characteristics of the prints. Biomaterials such as hydroxyapatite [235,236], polycaprolactone [237,238,239], poly-L-lactic acid [240,241], or collagen [242,243,244] are great candidates to replace or to be added to GelMA multicomponent hydrogels.

However, materials that exhibit enhanced mechanical properties could affect the viability of stem cells or the efficiency of the printing process [245]. A solution to balance the challenges of using stiff biomaterials could be the design of scaffolds with numerous pores, favoring the proper development of stem cells, while also allowing for the development of optimal vascularization.

As mentioned earlier, treating patients with dental implants without addressing the associated periodontitis is highly inefficient. Defense mechanisms against oral bacteria are impaired if the periodontal ligament is missing [246]. A solution to this issue is the coating of dental implants with human periodontal ligament stem cells. It was shown that these stem cells perform well on titanium dental implants, a material with good properties when used in dental applications [174,246]. The periodontal ligament stem cells promote the generation of fibrous tissues, cementogenesis, and enhanced aesthetics [246]. Human periodontal ligament stem cells also demonstrated good results, and 3D-bioprinted tissue models of the alveolar bone–periodontal ligament biointerface showed enhanced physical, chemical, and rheological properties [247].

One of the most recent approaches in tissue engineering and regenerative medicine is the development of extracellular vesicles, also known as exosomes. Usually referred to as a “cell-free” tissue engineering method, when bioprinted, it can potentiate stem cells in the printed scaffold [248,249]. Bioprinting exosomes has also been used in the treatment of periodontal defects, with the main advantage being the enhancement of stem cell activity without modifying the mechanical properties of the final product [250].

While the repair of periodontal tissue defects using 3D bioprinting has shown promising results, translation to human patients has not yet been achieved. Mechanical stability of the scaffolds remain a critical issue to be considered in parallel with the enhancement of stem cells’ period of activity. Additionally, scaffold vascularization optimization is essential, especially for periodontal ligament scaffolds, where metabolite flow is crucial. Further development and testing of bioinks in human patients are necessary for implementing the tissue engineering results presented above.

### 2.8. Salivary Glands 3D Bioprinting

The parotid, submandibular, and sublingual glands, along with the numerous minor salivary glands found in the oral mucosa, ensure many functions such as antimicrobial activity, and facilitating swallowing, mastication, and speech [251,252]. Many pathologies (autoimmune disorders—Sjögren’s syndrome, sialadenitis) or therapies (either medication-based or radiation therapy) can lead to xerostomia, which seriously affects the patient’s quality of life [253,254,255]. Traditional treatments for xerostomia in patients include the use of pilocarpine to stimulate saliva production and surgical interventions to unblock the main ducts. In more severe cases, when gland resection is performed, artificial saliva substitutes are prescribed to improve xerostomia symptoms [256,257].

It can be noticed that none of these common therapeutic approaches for various salivary gland defects attempt to restore the internal architecture or functions of the glands. Self-regeneration in these structures is limited in most adults [258]. In recent years, stem-cell therapies have been attempted with partial success. While salivary gland stem cells can help restore the functionality of the affected areas, there is still an inability to control cell placement, thus resulting in a chaotic architecture [258]. There is a need for regenerative approaches in which good architecture and functionality can be achieved, and an innovative approach is via 3D bioprinting.

Studies on the value of the elastic modulus of the major salivary glands in humans indicate that the optimal interval of functionality is at around 10–20 kPa. These values are required for maintaining the integrity of the acinar structures of the glands, while also not collapsing under the muscular tension involved in the mastication process [259,260,261]. Therefore, besides ensuring biocompatibility, functionality, and bioactivity of the salivary scaffold, an important characteristic is reaching these mechanical values without affecting the process of 3D bioprinting, nor the viability or proliferation of the stem cells used in bioprinting mixtures.

GelMA is a strong candidate for creating the microarchitecture of salivary glands that ensures the functionality of a gland equivalent. While mimicking the microenvironment, it also offers a favorable mechanical balance, an optimal stiffness that can be adjusted by changing the concentration of methacryloyl added in the hydrogel, and an optimal elastic modulus ranging from 1 to 20 kPa, suitable for the acinar and ductal system of the salivary glands [262]. A recent study used GelMA and alginates to further increase the mechanical stability of the bioink in the process of printing, aiming to create an innovative coaxial design with intersecting hollow tubes. This design allowed the primary salivary gland cells derived from mouse salivary glands to create acinar-like spheroids with enhanced functionality [262].

In theory, as long as the mechanical properties of different biomaterials are achieved without affecting printer functionality or cell viability, any substance can be used for these applications. Thus, other bioactive materials could serve as promising candidates: hyaluronic acid-based hydrogels, which present good mechanical stability for acinar-like structures [263]; polyethylene glycol hydrogels, which offer tunable mechanical properties while promoting cell viability [264]; or fibrin hydrogels, with tunable elastic properties, and the ability to promote cell migration and adhesion [265].

Another study used gelled egg yolk plasma, a natural compound, as a gel for salivary gland scaffold generation [266]. Similarly to the other hydrogels mentioned, its role is to mimic the natural ECM of the salivary glands. Rich in vitamins, lipoproteins, and ECM-like proteins, egg yolk plasma is a good candidate from a bioactive standpoint. Although its mechanical characteristics were not reported, based on the proteins of egg yolk, the elastic modulus is estimated to range between 1 and 5 kPa, which could be a suitable value if further enhanced with other materials or by adjusting the thermal gelation process [266,267]. Compared to other materials, an advantage of using egg yolk plasma is that it contains natural growth factors such as TGF-β, which can promote cell proliferation and viability within the bioink mix; in this case, salivary epithelial cells were used [266,268].

The stem cells used in the aforementioned studies were human or animal salivary gland stem cells. However, other candidates exist for simulating acinar-like structures, such as dental pulp stem cells (DPSCs). It has been observed that when DPSCs are exposed to fibroblast growth factors, epidermal growth factors, and bone morphogenetic proteins, they undergo acinar-like differentiation [269]. While these cells have only been tested in animal models, they could represent a good alternative for gland tissue engineering and may be further integrated into various hydrogels such as GelMA, HA, or fibrin hydrogels, as reviewed earlier.

Although the outlook of regenerative medicine for salivary glands is promising, an entire gland has yet to be created. Ensuring good vascularization of these structures is essential for proper salivary secretion and the flow of metabolites and nutrients, which also enhance cell viability. Furthermore, hybrid salivary glands would require neural control and communication between the major glands through neural and hormonal signaling [258]. The incorporation of different neovascularization factors into the prints or the design of prevascularized scaffolds could be an achievable approach in the near future, but designing appropriate neural control mechanisms requires further study.

### 2.9. Skin and Hair 3D Bioprinting

The significant progress of 3D bioprinting technology may enable the creation of a bionic head and neck complex. Numerous pathologies—such as thermal or chemical burns, traumatic events, or even genetic disorders—severely impact patients, and restoring aesthetics and functionality, including facial expressions or other receptive functions, remains highly challenging [270,271].

Skin grafts remain the gold standard for restoring skin integrity. However, the lack of sweat gland function, neural control, and hair follicles, as well as the presence of the immune response against the graft or associated infections, represent major disadvantages of this method [272]. Through 3D bioprinting, multi-layered printing has become more controlled, allowing for a better balance in the cell–biomaterial arrangement and advancing the regenerative aspects of skin treatment [273,274].

**Figure 3:**
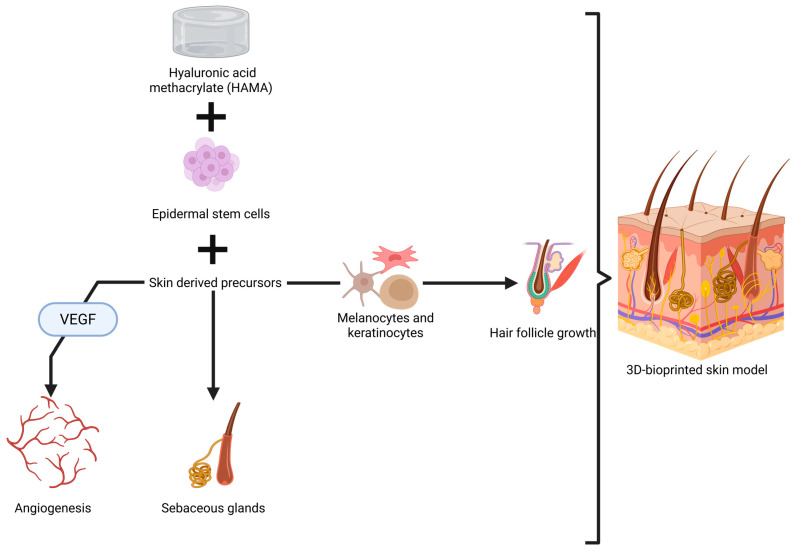
Visual representation of 3D printing of skin with sebaceous glands and hair follicle growth [275,276,277]. Created in BioRender. Timofticiuc, I. (2025) https://BioRender.com/kt50osr. Date of last access—21 June 2025.

An important achievement in this direction is represented by a bi-layered skin model tested in nude mice. Epidermal stem cells and skin-derived precursors were added to properly stimulate hair follicle regeneration and sebaceous gland formation, as well as to to promote angiogenesis [275]. These cells were added into a multi-hydrogel mix made of GelMA and hyaluronic acid methacrylate (HAMA), chosen for its mechanical strength and elastic properties [275]. While the results are excellent, especially due to the capacity of the skin model to allow functionality and formation of glands and hair (Figure 3), some aspects need to be discussed regarding its application in humans, especially in the head and neck area.

Ma et al. ensured mechanical strength by adding 0.5% HAMA to the bioink; however, the elastic modulus and other mechanical characteristics were not reported [275]. We need to consider that the average range of skin stiffness is 13.2–33.4 kPa, which varies greatly in different areas of the body [278]. For example, the chin has a low stiffness of around 20 kPa with a high percentage of elasticity (50.6%), while the back of the ear presents a stiffness of 40 kPa [42]. Furthermore, there are different mechanical properties between each layer of the skin; the dermis has a Young’s modulus of around 40 kPa, while the epidermis can reach up to 4 MPa [279].

Furthermore, unlike other regions, the skin of the head and neck not only requires functionality, mechanical strength, and elasticity, but also a cosmetic appearance, which can be difficult to control in models where stem cells activate independently. In addition, the growth of facial hair is different from other parts of the body [280]; thus, creating models for this area is more challenging. Neovascularization in these artificial models may be improved by adding VEGF, or incorporating other stem cells and growth factors—potentially representing a promising direction for future research.

Other scaffolds have also been designed, and improved methods for controlling hair follicle formation and hair growth have been identified, such as the use of mesenchymal stem cells [281]. Through the combination of epithelial and mesenchymal stem cells with collagen droplets, a model with a high density of cells was obtained [281]. This promoted small contractions in the collagen hydrogel, mimicking the natural tensions found in tissues. This characteristic supports better development of integrative elements of the models, such as vascularization or ECM [281].

Another study highlights the importance of incorporating other types of stem cells for adequate hair follicle generation, such as keratinocytes and melanocytes. These cells contribute to the consolidation of the hair follicle while also playing a role in hair pigmentation, making the skin models more realistic and aesthetic [282]. Integrating sweat gland stem cells in skin models is also possible, which completes the elements required for an aesthetic and functional skin model [283]. More efficient methods of printing, such as robot-assisted techniques, have also been tested when building models based on epidermal and mesenchymal stem cells [284,285].

Controlling such a high number of stem cells in a model is very difficult, especially due to their high metabolite production, which requires good vascularization. Moreover, studies investigating their interactive behavior for long periods of time are lacking, so aspects regarding the immunological response and resistance over time should be further researched. The subcutaneous tissue also warrants further investigation, and some promising results have already been reported using adipose stem cells for generating this layer [286]. However, additional studies may reveal the complexity of creating multilayered models, while also preserving the aesthetic aspects and different mechanical characteristics of each area of the head and neck.

### 2.10. Exploring the Feasibility of a Bionic Neck

A bionic neck would require crafting a model mimicking all the functions of the neck, while also ensuring integration with other anatomical regions in terms of vascularization, neural control, endocrine control, and muscular and osteoligamentous integration for stability and structural integrity. We have addressed the soft and functional components of this region, i.e., the functions performed by the larynx, trachea, pharynx, the initial part of the esophagus, and also the endocrine elements: thyroid and parathyroid glands [5]. However, the number of bioprinting studies in this anatomical area is very limited. Except for the larynx and trachea, where some research has been conducted, other elements of the neck have not yet been properly studied regarding bioprinting methods, alternative regenerative treatments, or integration into human patients.

### 2.11. Larynx 3D Bioprinting

The larynx presents a complex neuromuscular architecture that balances its vital functions: the passage of air, protection of the airway during swallowing, and phonation, the latter standing out due to its psychological and social implications [287,288,289]. Many pathologies, either congenital malformations or acquired defects from traumatic events, inflammation, cancer, or allergic conditions, can affect the larynx’s capacity to perform its critical functions [290,291,292,293].

Depending on the pathology affecting the larynx, many treatments can be performed, but each one presents challenges and room for improvement. Muscle-related defects can be treated pharmacologically, but the uncertainty regarding the administered dosages and the frequent dysphagia as a side-effect calls for an alternative to be sought [294,295]. Surgical reconstruction of the larynx can also be performed, but many of the interventions are associated with increased complication rates, prolonged healing, and high mortality [296,297]. Furthermore, nerve-related defects, which often result in voice loss, can be treated using recent reinnervation techniques, but with high risks and often yield only partial restoration of phonation [298,299].

The main areas of progress in 3D bioprinting of larynx models have been in relation to recreating its complex structures. Maintaining cell viability in the scaffold while ensuring the scaffolds have good mechanical strength represents a major challenge. The structure of the larynx is firm, but increasing the rigidity of the bioinks requires higher concentrations and viscosities of the hydrogels [300]. The study performed by Galliger et al. demonstrated how cooling systems can increase the mechanical characteristics of the final print required for a larynx equivalent, without affecting the printing process [301]. Additionally, the team developed a mix using decellularized extracellular matrix microparticles of porcine origin to further optimize the printability at lower temperatures, of around 15˚C.

Although GelMA or any other methacryloyl-based bioinks allow a high rate of cell viability post-printing (around 90%), in the study of Galliger et al., no cells or growth factors were used [23]. Obtaining superior mechanical properties and optimizing printing techniques for a simpler print process are promising developments; however, it is unclear if increasing internal forces of the scaffold to better mimic the larynx structure is supported by the cells. Furthermore, increasing the concentrations of the materials used in the scaffolds may decrease pore size, negatively impacting cell viability and function [300].

Other methods have also been proposed to meet the mechanical needs of an artificial larynx model. By integrating a mix of hydrogels (GelMA-glycidyl-methacrylated HA) in a polycaprolactone scaffold, increased structural support can be achieved [302]. By adding a biopolymer such as PCL, a controlled and prolonged degradability of the scaffold is ensured [174,302]. This aspect allows sufficient time for the added stem cells to develop and proliferate in the scaffold, while also replacing the PCL. Over time, a natural chondrocyte mechanical structure that supports the hybrid hydrogel scaffold is created, offering improved integration of the model into a potential patient [302].

However, as observed in the aforementioned study, a larynx model was built only focusing on its cartilaginous constitution. Musculoskeletal differentiation is also important if an accurate model is desired, and this can be achieved by introducing bone marrow-derived mesenchymal stem cells, as shown by McMillan et al. [303]. This reiterates the idea of more in-depth research on the generation of multilayer structures in which superior control can be achieved in terms of cellular composition and mechanical properties.

Features such as clinical translation, scaling up models for integration in human patients, the viability of the cells, and identifying what types of cells better perform under mechanical conditions as found in the larynx, need to be addressed in future research.

### 2.12. Other Neck Elements and Future Possibilities

Anatomically, the larynx is formed of cartilaginous components (thyroid, cricoid, and arytenoid cartilages) supported by ligaments and muscles [304]. The trachea is also formed of cartilaginous structures, differently shaped to ensure airway passage, mobility, and elasticity during different neck movements or swallowing [305]. Thus, for the cartilaginous part of the trachea, a similar approach to that of the larynx can be undertaken [306,307].

Including chondrogenic stem cells in the model and using compatible hydrogels or polymers with superior mechanical properties could lead to great results. Several studies have explored cartilage and smooth muscle formation for engineering tracheal tissue [308]. A polycaprolactone scaffold with added human mesenchymal stem cells was used to create a trachea model with cartilaginous and muscular components that presented mechanical properties similar to the physiological ones [308].

Hydrogel mixes have also been designed to mimic the extracellular matrix of the trachea, ensuring good chondrogenic differentiation. An innovative approach for bioprinting soft tissues was represented by a bioink made of methacrylated HA, polyethylene glycol succinic acid ester (PEG-NHS), and methacryloyl-modified dermal acellular matrix, which promoted vascularized fibrogenesis, thus addressing vascularization—one of the main drawbacks [309]. Adding fibroblast and chondrocyte stem cells to this mix resulted in obtaining a perfect balance, leading to the formation of mechanically stable C rings and a functional membranous structure, i.e., the vascularized fibrous rings [309].

Furthermore, through chemical reactions obtained by adding PEG-NHS, a stable bond was formed between these rings, achieving an anatomical tracheal equivalent [309]. However, translating these results to humans presents a high risk, mainly due to the need to ensure good airway passage. Moreover, functionalizing the trachea has not yet been achieved, and this could represent the next objective for future research in airway passage bioprinting.

The esophagus and the pharynx are mainly formed of smooth and skeletal muscle [310,311]. Without taking into consideration the neural control that needs to be obtained in these organs, and their secretory functions [310,311], the main objective of research in 3D bioprinting models for these organs should be obtaining a muscle-like scaffold with regulated contraction. Hence, compatible materials and optimal development of stem cells need to be investigated. Some elements have already been studied, but not specifically for the pharynx or esophagus. For example, microfluidic 3D bioprinting was used to ensure good control over cell placement, allowing for human smooth muscle cells to be bioprinted in a scaffold, and controlled contractions and pharmacological response were obtained [312].

Other types of stem cells can be targeted in the formation of smooth muscle using a decellularized smooth muscle extracellular matrix as a microenvironment for stem cell development [313]. Using this approach, adipose-derived mesenchymal stem cells encapsulated in a hydrogel were able to express smooth muscle proteins (alpha-actin) in a 3D bioprinted scaffold [313]. This is a promising result demonstrating various possibilities for generating smooth muscle from different stem cells, while also addressing the regenerative and contractile properties of 3D bioprinted smooth muscle scaffolds.

Through similar approaches, skeletal muscle can also be generated. Scaffolds with contractile and pharmacological responses were crafted using primary human muscle precursor cells, carefully printed after being coated with hydrogels for mechanical stability and protection of the cells during printing [314]. Results on murine models are also promising, providing support for the translation into human models. A team of researchers managed to apply murine muscular stem cells to generate an entire tibial muscle, which was later grafted into the animal model [315]. Worth mentioning is the use of polymers (PEG) instead of hydrogels for the generation of a mechanically stable scaffold for this model, demonstrating the versatility of biomaterials that can be used for future medical applications [315].

These few studies mentioned above are proof enough that smooth and skeletal muscles can be bioprinted. However, integration of these constructs into a muscle-based organ model is challenging. It is still uncertain how the stem cells added to the bioinks integrate with other elements of an active organism. Their activity, viability, or functionality is not fully controllable. Furthermore, a self-sustainable cycle of life for these scaffolds is not yet ensured because the results do not show how these scaffolds or models act in prolonged use. Additionally, neural control is not easily replaceable using only pharmacological approaches or one-function scaffolds. Metabolite production could cause critical defects in the scaffolds or produce immunological or allergic responses in patients if not controlled correctly under optimal vascularization. These are critical aspects that researchers should take into consideration.

Generally speaking, ensuring functionality represents one of the most challenging aspects of artificially created models or scaffolds. It is uncertain whether the use of stem cells derived from donor organs or tissues is sufficient for generating 3D builds capable of bioactivity in the replicated structures. Both mechanical characteristics and cell viability are critical coordinates when first designing such constructs.

While the possibility of generating a bionic part of the human body exists, it is mandatory to properly document all the potentially obtainable bioinks to reach this ambitious medical objective. Developing more materials, researching the maximal viability of the added stem cells, finding solutions to ensure the cells’ cycle of life, and controlling their development are some of the main directions that should be researched in this field.

## 3. Vascularization Possibilities and Neural Control for 3D Models

The rapid development of materials and 3D bioprinting techniques has led to impressive achievements in creating patient-specific and self-regenerative models or scaffolds. However, the dimensions of scaffolds have rapidly scaled up, leading to challenges in their functionality [316]. One of the significant issues when incorporating cells into these constructs is the need for nutrients and oxygen, necessary for cell viability and development [316].

When small scaffolds, under 0.2 mm in thickness, were initially designed for in vitro testing, the nutrients in the in vitro microenvironment were sufficient to supply the cells with all requirements [317,318]. Due to upscaling, the main concern in recent studies has been the identification of methods to prevent the necrosis that occurs frequently in large and thick scaffolds [317,318].

Various solutions have been proposed to enable metabolite and nutrient flow in 3D bioprinted constructs. One approach relies on building vascular networks in vitro, thus creating pre-vascularized scaffolds to be later grafted and connected to the patient’s blood supply. For this purpose, a more precise bioprinting technique called microscale continuous optical bioprinting needs to be employed, creating very high-resolution scaffolds that permit adequate vessel development [319].

Another avenue involves printing endothelial cells directly into the scaffold at the same time as the hydrogel, spontaneously generating in vitro tubular-like structures with the ability to connect to the patient’s blood supply when transferred in vivo [319]. Designing pores in the scaffolds facilitates optimal development of the endothelial cells, thus resulting in improved release of VEGF. An optimal prevascularized scaffold can be obtained through this method by creating a ceramic outline, which will result in high water retention—necessary for good cell development—with pores ranging from 80 to 100 µm [320].

Creating pre-vascularized scaffolds does not require, in all cases, the use of stem cells. If more specific and controlled vascular networks are desired, direct printing can be performed using a bioink that will be later removed, also known as sacrificial ink [321]. In the end, an exact tubular-like network is created that can play the role of a vascular network equivalent.

Prevascularized scaffolds can be safe for patients in some aspects. The initial vascular formation is created in a controlled in vitro environment, so any potential defects are easy to observe, and also, prolonged viability tests can be performed. A disadvantage of adopting this solution could be the long period required for the production and testing of the scaffold.

Other studies rely on introducing angiogenesis factors and stem cells that promote vessel formation in the scaffold, in vivo. Bioprinting a co-culture of human adipose tissue-derived microvascular fragments and human dermal fibroblasts can lead to an in vivo self-assembled capillary network that connects to the host’s blood supply [318]. Additionally, vascular growth factors can be directly added to the scaffolds to support the formation of vascular networks, enhance stem cell activity, and ensure a more controlled development of vessels [322].

**Figure 4:**
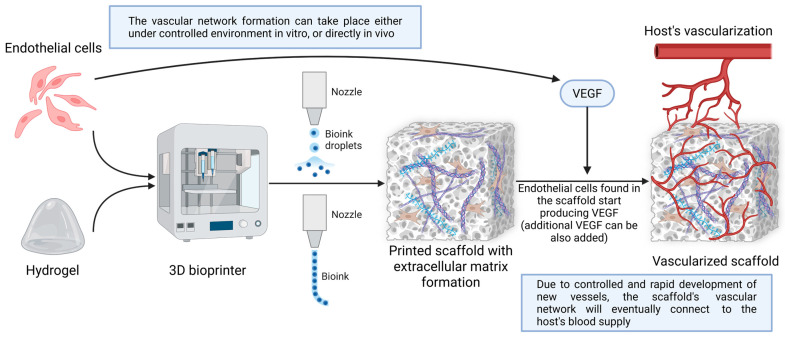
Method for the vascularization of 3D bioprinted constructs. In the initial stages, optimal cells are selected to formulate a bioink based on hydrogel or other materials. Next, through different bioprinting techniques (either droplet-based or classic extrusion), a scaffold or model is obtained, in which extracellular matrix elements form due to the intrinsic activity of the introduced materials and cells. In this case, endothelial cells produce VEGF, a growth factor that underlies the formation of new blood vessels. This process can be controlled in vitro or left to self-regulate in vivo. Finally, the scaffold or model is transferred to patients, where it ends up connecting to the host vasculature. Created in BioRender. Timofticiuc, I. (2025) https://BioRender.com/bintbnp. Date of last access—3 May 2025.

Many approaches to vascularizing 3D bioprinted constructs exist (Figure 4). The best method to vascularize bioprinted models needs to be chosen in order to best fulfill its role in patients.

Of course, in most tissues, innervation and associated neural control are of vital importance; the adverse effects of nerve damage have been demonstrated in numerous cases, such as nerve-destroying infectious agents [323,324,325]. To perfectly complete the intrinsic control of tissue-engineered scaffolds, neural control is the final piece to fully mimic a human tissue or organ, at least from a mechanical functionality point of view. However, studies towards this complex achievement have mainly focused on stimulating the proliferation of neural cells in the scaffolds and designing neural networks capable of responding to chemical or electrical stimuli.

In the study of Joung et. al, neural activity was obtained after neural progenitors were added to the bioinks. After successful printing, these cells were able to differentiate into active neurons [326]. Similar results were obtained by adding pluripotent stem cells to the bioinks, where populations of neural cells with the desired functional characteristics were obtained [327].

More recent studies have managed to create neural tissues with enhanced neural activity and functional neural connectivity. These results were obtained after adding Neurogenin-2 human pluripotent stem cell lines to the hydrogel-based bioinks [328,329]. It was observed that by simply adding specific stem cells, theoretically, any function can be achieved. By respecting the mechanical aspects supported by these cells and printing them in compatible biomaterials, any function can be obtained. Regarding further steps in integrating neural scaffolds into patients—especially brain scaffolds—one of the main challenges is blood supply integration. A simple neovascular proliferation is not sufficient due to the complex vascular network of the brain [330].

However, controlling cellular development, increasing viability, and achieving full functional control remain challenges that need to be further addressed. Neural control is not limited to applying stimuli to the scaffold to obtain any kind of response. Ideally, achieving neural control in bioengineered scaffolds should mean establishing connectivity of neural networks with host nerves, ensuring hierarchical control over the constructs. All these aspects are to be researched in future studies.

## 4. Summary on 3D Bioprinting Technologies

The main aim of this paper is to review 3D bioprinted soft tissue scaffolds with potential for translation into clinical practice. Most of the constructs presented here are created using extrusion-based bioprinting. There are a few advantages associated with this technique, such as the possibility of printing very high cell-density scaffolds, using various types of different cells, thus allowing for the production of complex cell architectural models [331].

Furthermore, there are various categories of biomaterials compatible with this kind of printing, which can be adapted for the necessities of the cells and the function they will perform [332]. However, these printers can produce high pressures in the moment of the extrusion, generating shear stress that may affect cell viability [331]. Other technologies exist, such as inkjet bioprinting, which is more precise, but are limited to low-viscosity bioinks [333,334].

A distinct group of 3D printing technologies is based on vat-photopolymerization, a group of techniques (DLP and SLA) that uses light to polymerize biocompatible materials [174]. These methods are known for very fast printing, with high precision and resolution, which can be used for printing bioinks; however, additional post-processing steps, such as UV light curing, are often required [174,335]. The main advantages derive from the smooth surfaces that these techniques can produce, and the speed with which this mechanical characteristic is obtained [336,337]. The smooth surfaces and the precisely controlled pore sizes are essential for good cell adhesion and functionality [336,338]; however, the necessity of UV post-processing can affect cell viability [339,340,341]. Furthermore, with this group of techniques, not many materials are compatible with the added cells [335,342].

These classic printing methods are constantly being developed to expand the range of printable materials, allowing for smaller printed structures, faster printing times, and increased cell viability. Future research will also explore new 3D printing techniques such as in situ bioprinting, which is based on the principle of using bioink directly at the site of the defect [343,344]; multi-material techniques, which allow the creation of highly complex scaffolds [345]; and AI-assisted bioprinting for improved control and precision [346]. Droplet bioprinting using an air-focused platform also shows promise in various medical applications [347,348]. Direct sound printing is another emerging technology that uses ultrasounds to create acoustic cavitation, enabling in situ sonochemical reactions and highly complex prints—even under the skin [349,350].

## 5. Challenges and Future Perspectives

While it is true that 3D printing has been gaining traction as a realistic therapeutic modality in medicine over the last decade, its regular everyday application has faced a series of bottlenecks [351,352]. Cell viability in the final biomaterial scaffold remains one of the main aspects that need to be addressed. Most of the time, the mechanical characteristics of the build affect the structural integrity of the cells. Shear stress generated by high concentrations of biomaterials such as gelatin drastically decreases the viability of cells—from over 60% to under 20% (at 10% *w*/*v* and 20% *w*/*v* of gelatin, respectively) [353].

In general, an acceptable cell viability is considered above 70%; thus, enhancing 3D bioprinting techniques and adapting biomaterials are key aspects that every study should consider [354]. Ways of adapting mechanical aspects of the build to maintain high cell viability include increasing pore sizes or decreasing the concentrations of the biomaterials, thereby reducing mechanical stress. However, this also reduces the mechanical integrity of the final build, greatly impacting its functionality [354]. There are other methods to modulate the increase in cell viability, either by incorporating cells into different substances that can have a protective effect, such as graphene oxide [355], or by adapting the extrusion pressure, lowering it to protect the cells from mechanical stress, or even by using special bioinks that generate low shear stress [356].

One of the least studied aspects of cell viability is the maximum time of their survivability. There are optimistic results regarding high rates of cell viability, such as fibroblasts with over 80% viability 48 h post-printing [357], and with prolonged periods of survival, for example, keratinocytes, which presented a few morphological changes 21 days post-printing [358]. However, when translating these results in clinical practice, where a patient-specific scaffold should function for at least a few years, it is uncertain how these cells will perform. As far as we know, there are no reported studies that consider cell viability after 1 year, but considering the emerging ideas on vascularization aspects presented above, optimistic results are expected.

Translating these results on bioprinted scaffolds into clinical practice not only requires an understanding of the period of time during which the added cells can perform, but also how they could possibly interact with the human body, and if they generate any kind of immunological response. Most of the applied cells in the above-mentioned studies are known for their low immunological response [359]. However, some studies report that the mechanical characteristics of the final build, particularly its non-viable aspects, can trigger a nonspecific immunological proinflammatory response (TNL/NF-κB; IL-6/JAK-STAT) [360]. Ensuring adequate vascularization of the build may reduce the immune response [361].

Until recently, only a few clinical studies had examined the usefulness of 3D printed biomaterials in therapy [362]. After the initial successful results, the aim was to implement these techniques into personalized care schemes. In the current setting, point-of-care 3D printing has garnered significant impetus in favor of its implementation [363].

Organizing clinical trials for 3D printing approaches, let alone their regular everyday use, presents considerable challenges. In the last decades, there has been a constant drive for innovation in treatment schemes and solutions [364], which has led to the rapid evolution of the regulatory aspect concerning clinical trials, both from a legal and ethical standpoint [365,366]. However, the introduction of 3D bioprinting is associated with a host of gaps and uncertainties [367,368]. In the pre-clinical bioprinting stage, the results in various applications are encouraging [369,370,371], and bioprinted constructs have also been used successfully in a research capacity [372,373,374]. Due to their very nature, 3D printed biomaterials of any type and application are subject to particular constraints and considerations [363]. The two relevant EU regulations are the EU Clinical Trials Regulation [375] and the Medical Devices Regulation [376], which safeguard the rights of the participants and ensure the best possible clinical practices [14]. Nevertheless, given the legal and legislative intricacies associated with 3D printed biomaterials, there are a number of points that must be taken into account [14].

The recent development of materials suitable for 3D printing [377,378] opens even more possibilities for novel approaches both from a research and a treatment perspective [379]. Some interesting future research perspectives may thus be offered. To begin with, as is the case with most artificial implants and materials, there is an increased risk of infection [380,381]; therefore, there is the possibility of natural compound integration with antimicrobial action, aside from antibiotics. Currently, treatment modalities in peri-prosthetic infections center around different antibiotic delivery modalities [382,383]. Natural compounds with multiple different actions, such as kaempferol [384,385], capsaicin [386,387], curcumin [388,389], pinosylvin [390,391], piperine [392,393], and thymol [394,395], could be added during the 3D printing process to reduce the chance of infection or excessive inflammation. Another avenue open is the use of artificial intelligence both for the identification of potential infectious complications [396,397]; however, there still remains a host of other issues to be resolved, including patient rights, privacy, and ethical considerations [398], along with issues pertaining to the issue of Big Data in medicine [399], which is necessary for such applications [400].

Looking ahead, studies should be performed to integrate bioprinted scaffolds first into animal models (in vivo) before translating them to human patients. These studies should follow several important directions: 1. the rate of neovascularization and the density of the vascularized network; 2. how future generations of cells from the initial cell load-up perform (morphological aspect, functional aspects, immunological response, structural integrity, localization in the scaffold), and how many generations of cells can be produced until the effectiveness of the scaffold drops significantly; 3. The long-term degradability of the biomaterial scaffold (e.g., after 1 year, after 5 years); 4. the possibility for revision surgeries (especially if the patients are young).

## 6. Conclusions

The main directions outlined in this paper relate to the possibility of creating bionic parts in the future. Existing approaches to 3D bioprinting of human components could allow the replacement of traditional treatments and benefit patients. Incorporation of the results in each anatomical component of the head and neck offers researchers a broad vision of what has been achieved and what remains to be researched in order to achieve the ideal of the medical realm, creating a bionic head or neck.

There are numerous challenges to the present scaffolds or models, but possibilities to overcome them have been proposed. The current paper describes the elements that these scaffolds require to be considered a complete equivalent of a certain human part. Furthermore, we described the complexity that needs to be achieved to perfectly mimic a human anatomical component. It is not necessary to create a complete functional replica to obtain great results, since the new constructs already expand numerous limitations of pre-existing treatments.

The promising results described in recent research need to be considered for translation to human patients. These solutions should be accessible, and their advantages should be clearly presented to medical teams and patients alike. Scaling up these models and researching methods to increase the viability of the constructs are critical aspects for the sustainability of these new achievements.

## Figures and Tables

**Table 1:** Mechanical properties of the natural human tissues and organs of the head and neck.

Physiological Tissue/Organ	Young’s Modulus (Stiffness)	Tensile Strength	Reference
Cornea	Central Cornea: 70–100 kPa Peripheral Cornea: 30–60 kPa	380–650 kPa	[27,28]
Retina	431 kPa	100 kPa (porcine models)	[29,30]
Choroid	4620 kPa (4.62 MPa)	300 kPa	[29,31]
Sclera	Anterior Sclera: 17,000–43,000 kPa (17–43 MPa) Equatorial Sclera: 9000–25,000 kPa (9–25 MPa) Posterior Sclera: 7000–18,000 kPa (7–18 MPa)	Not mentioned	[32]
Ear Auricle	4500–5900 kPa (4.5–5.9 MPa)	3460 kPa (3.46 MPa)	[33,34]
Tympanic Membrane	20,000–40,000 kPa (20–40 MPa)	25,000–32,200 kPa (25–32.2 MPa)	[35,36]
Nose	2100–3300 kPa (2.1–3.3 MPa)	1640–2140 kPa (1.64–2.14 MPa)	[37,38]
Periodontal Tissue	10–31 kPa/900–1200 kPa	Not mentioned	[39,40]
Salivary Glands	Parotid glands: 18.4 kPa Submandibular glands: 15.9 kPa	Not mentioned	[41]
Skin	Back of the ear: 40 kPa Chin: 20 kPa	Not mentioned	[42]
Larynx	8.6 kPa	1000 kPa (1 MPa)	[43,44]
Trachea	1000–15,000 kPa (1–15 MPa)	1200–2500 kPa (1.2–2.5 MPa)	[45,46]
Esophagus	Not mentioned	1200–2800 kPa (1.2–2.8 MPa)	[47]

**Table 2:** Mechanical properties of the hydrogels and other biomaterials that could be used as scaffold materials for different medical applications.

Material	Young’s Modulus (Stiffness)	Tensile Strength	Advantages	Disadvantages	**Refs**
**Hydrogels**					
Alginate	<1.5 kPa	Up to 1830 kPa (1.83 MPa)	-Ease of gelation; -Low shear stress; -Compatible in mixes with other materials;	-Low mechanical strength; -Cytotoxicity; -Low cell adhesion;	[48,49,50,51,52,53,54]
Gelatin	81 kPa	24 kPa	-Bioactive; -Thermosensitive; -Low shear stress;	-Low mechanical strength; -Thermal instability;	[55,56,57,58,59,60,61]
GelMA (Gelatin Methacryloyl)	29.2–43.2 kPa/Up to 200–1000 kPa (Influenced by the % of methacrylation)	2800–3800 kPa (2.8–3.8 MPa)	-Photopolymerization (suitable for DLP- or SLA); -Adjustable mechanical properties (different concentrations of methacrylate can be added); -Great support for cells;	-Low viscosity; -Sensible to the microenvironmental conditions; -Cytotoxicity; -Limited mechanical stability;	[62,63,64,65,66,67,68,69,70]
Collagen	120–250 kPa	40 kPa	-Good cell adhesion; -Good mechanical properties, which can be adjusted with various methods of crosslinking;	-Low mechanical strength; -Fast degradation rate (this could also represent an advantage if used in fast-degradable scaffolds); -Thermal instability;	[60,64,71,72,73,74,75,76]
Fibrin	15–150 kPa	1.6–10 kPa	-Cell migration and adhesion; -Adjustable mechanical properties; -Mimics the natural extracellular matrix;	-Fast degradation rate (this could also represent an advantage if used in fast-degradable scaffolds); -Costly; -Sensible to the microenvironmental conditions;	[77,78,79,80,81,82,83]
Hyaluronic Acid	24 kPa	63 kPa	-Wettability; -Dynamic cross-linking; -Promotes cell adhesion, cell viability, and cell mobility;	-Costly; -Limited mechanical properties; -Sensible to the microenvironmental conditions;	[84,85,86,87,88,89,90]
Chitosan	7900–92,000 kPa (7.9–92 MPa)	3600–12,100 kPa (3.6–12.1 MPa)	-Easy gelation; -Bioactive; Adjustable mechanical properties;	-Limited printability; -Hard to obtain from natural sources; -Limited printability; -Limited mechanical stability;	[91,92,93,94,95,96]
Agarose	100–300 kPa	Not mentioned	-Exhibits rheological properties; -Thermosensitive; -Promotes cell viability;	-Limited mechanical stability; -Sensible to the microenvironmental conditions; -Costly;	[97,98,99,100]
Silk Fibroin	Up to 6300–6700 kPa (6.3–6.7 MPa)	80–700 kPa	-Good mechanical properties; -Promotes cell adhesion, mobility, and viability;	-Different properties if extracted from different sources; -Sensible to the microenvironmental conditions; -Low wettability;	[101,102,103,104,105,106]
**Other Biomaterials**					
Polyethylene Glycol (PEG)	16.5–89.5 kPa	Not mentioned	-Adjustable mechanical properties; -High wettability;	-Limited cell adhesion; -Potential toxicity; -Costly; -Limited mechanical strength;	[107,108,109,110,111]
Polylactic Acid (PLA)	3.5 GPa (3500 MPa/3,500,000 kPa)	60,000 kPa (60 MPa)	-Thermal and mechanical stability; -Wettability (this could be so intense that it could represent a disadvantage)	-Costly; -Limited cell adhesion; -Fast degradation rate (this could also represent an advantage if used in fast-degradable scaffolds);	[112,113,114,115,116,117,118,119]
Polycaprolactone (PCL)	25,000–120,000 kPa (25–120 MPa)	Not mentioned	-Good mechanical properties; -Adjustable degradation rate; -Promotes cell adhesion and viability;	-Limited wettability; -Thermal instability; -Costly;	[120,121,122,123,124,125]
Poly (lactic-co-glycolic acid) (PLGA)	32,100–36,100 kPa (32.1–36.1 MPa)	1600–2000 kPa (1.6–2 MPa)	-Adjustable mechanical properties; -Adjustable release of various drugs;	-Hydrophobic; -Fast degradation rate (this could also represent an advantage if used in fast-degradable scaffolds); -It can generate inflammatory response;	[83,120,126,127,128,129]

## Data Availability

No new data were created or analyzed in this study. Data sharing is not applicable to this article.
